# Clinical significance of circulating microRNAs as diagnostic biomarkers for coronary artery disease

**DOI:** 10.1111/jcmm.14802

**Published:** 2019-11-11

**Authors:** Lei Zhang, Yuan Zhang, Sheng Xue, Han Ding, Yu Wang, Hongzhao Qi, Yin Wang, Wenjie Zhu, Peifeng Li

**Affiliations:** ^1^ Institute for Translational Medicine Qingdao University Qingdao China; ^2^ The Affiliated Hospital of Qingdao University Qingdao China

**Keywords:** circulating micrornas, combined diagnosis, coronary artery disease, diagnostic biomarkers

## Abstract

Coronary artery disease (CAD) is one of the biggest threats to human life. Circulating microRNAs (miRNAs) have been reported to be linked to the pathogenesis of CAD, indicating the possible role in CAD diagnosis. The present study aimed to explore the expression profile of plasma miRNAs and estimate their value in diagnosis for CAD. 67 Non‐CAD control subjects and 88 CAD patients were enrolled. We conducted careful evaluation by RT‐PCR analysis, Spearman rank correlation coefficients analysis, Receiver Operating Characteristic (ROC) curves analysis and so on. The plasma levels of six miRNAs known to be related to CAD were measured and three of them showed obvious expression change. Circulating miR‐29a‐3p, miR‐574‐3p and miR‐574‐5p were all significantly increased. ROC analysis revealed the probability of the three miRNAs as biomarkers with AUCs (areas under the ROC curve) of 0.830, 0.792 and 0.789, respectively. They were significantly correlated with each other in CAD patients, suggesting the possibility of joint diagnosis. The combined AUC was 0.915, much higher than each single miRNA. Therefore, our study revealed three promising biomarkers for early diagnosis of CAD. The combination of these miRNAs may act more effectively than individual ones for CAD diagnosis.

## 1 INTRODUCTION

Coronary artery disease (CAD) is a global health issue and a leading cause of the mortality. Some biomarkers were developed to improve the diagnostic efficiency, such as creatine kinase‐MB isoenzyme (CK‐MB) and cardiac troponin (TnT or TnI) proteins. Nevertheless, these protein biomarkers have several drawbacks.^1^ Thus, specific and reliable biomarkers for CAD diagnosis are in urgent need.

In recent years, circulating miRNAs (the miRNAs in plasma, serum and other body fluids)^2^, ^3^ emerged as promising and novel biomarkers for the diagnosis of CAD.^4^, ^5^ miRNAs are small (approximately 22‐nucleotide) non‐coding RNAs, participating in diverse physiological and pathological processes. miRNAs are key regulators in the function of cardiomyocytes, endothelial cells (ECs), vascular smooth muscle cells (VSMCs) and platelets which involve in the initiation and progression of atherosclerosis—the main cause of CAD. miR‐29a is required for normal function of endothelial cells and can restore it in cardiometabolic disorders.^6^ miR‐29a also takes part in the survival and function maintenance of cardiomyocytes.^7^, ^8^ Besides, miR‐29a plays an important role in cardiac tissue protection in stress condition.^9^ miR‐134 could be biomarkers for acute myocardial infarction.^3^ miR‐223 secreted by blood cells could enter vascular cells and walls and then play important roles in VSMC function and atherogenesis.^10^ miR‐574 shows increased expression in infarcted heart disease.^11^ miR‐574‐5p can promote VSMCs proliferation.^12^ miR‐765 plays a role in CAD diagnosis.^13^

In this study, we investigated the plasma levels of six plasma miRNAs (miR‐29a‐3p, 134‐5p, miR‐223‐3p, miR‐574‐3p, miR‐574‐5p and miR‐765). The miRNAs with obviously altered expression levels in CAD patients compared with controls patients were selected to explore the diagnostic value and evaluate the combined effect.

## 2 MATERIALS AND METHODS

### 2.1 Study participants

A total of 67 control samples and 88 CAD samples were enrolled in this study. The whole protocol of this study was admitted by the ethics committee of the Affiliated Hospital of Qingdao University.

### 2.2 Plasma collection and RNA isolation

Fast blood samples (~5 mL) were withdrawn in EDTA‐anticoagulated tubes, and the plasma (the supernatant) was collected by the centrifugation method.

Total RNA was isolated with the TRIzol extraction method. Glycogen at a final concentration of 0.1 μg/μL was added to increase the RNA yielding.

### 2.3 Detection and quantification of miRNAs by qRT‐PCR

miRNAs were converted into cDNA and quantified by quantitative real‐time polymerase chain reaction (qRT‐PCR). SYBR Green miRNA qRT‐PCR kits and a Bio‐Rad CFX96 system were applied. The primers and specific miRNA sequences are listed in Table S1. We used U6 snRNA as the housekeeping gene.

### 2.4 Statistical analysis

The distribution of miRNA expression was analysed by GraphPad Prism software. The differences in clinical characteristics between CAD patients and control patients were analysed by Mann‐Whitney *U* test. The correlation analysis was carried out by the Spearman correlation coefficient (GraphPad Prism software). The diagnostic values were evaluated by receiver operating characteristic (ROC) curves analysis (MedCalc software). The area under the ROC curve (AUC) was considered as a critical diagnostic index. All tests were two‐sided or two‐tailed. A difference with a *P* < .05 was statistically significant.

## 3 RESULTS

### 3.1 Basic clinical characteristics

**Figure 1:**
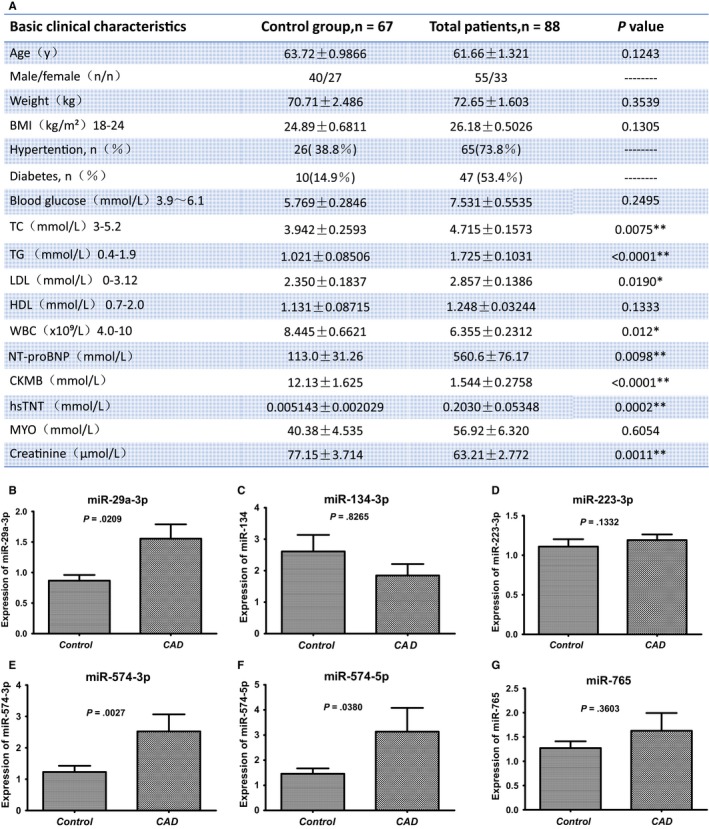
Clinical characteristics of the study population and plasma levels of circulating miRNAs. A, Baseline and clinical characteristics of the study population. BMI, body mass index; CK‐MB, creatine kinase‐MB; HDL, high‐density lipoprotein; hsTnT, high‐sensitivity troponin T; LDL, low‐density lipoprotein; MYO, myoglobin; NT‐proBNP, N‐terminal pro–B‐type natriuretic peptide; TC, total cholesterol; TG, total triglyceride; WBC, white blood cell. Data are shown as mean ± SEM; *P < .05 and **P < .01. B, Plasma levels of miR‐29a‐3p. C, Plasma levels of miR‐134‐3p. D, Plasma levels of miR‐223‐3p. E, Plasma levels of miR‐574‐3p. F, Plasma levels of miR‐574‐5p. G, Plasma levels of miR‐765. Expression levels of selected miRNAs were analysed by qRT‐PCR, and U6 snRNA was used as the reference gene. Data are presented as mean ± SEM

The clinical and laboratory characteristics were counted. Significance analysis was performed between the control group and the CAD group (Figure 1A).

### 3.2 Expression profiling of plasma miRNAs in CAD patients versus control patients

The expression patterns of six miRNAs (miR‐29a‐3p, 134‐5p, miR‐223‐3p, miR‐574‐3p, miR‐574‐5p and miR‐765) were investigated by qRT‐PCR. Out of the six miRNAs, plasma miR‐29a‐3p, miR‐574‐3p and miR‐574‐5p were up‐regulated in CAD patients compared with control patients (1.79‐fold, 2.05‐fold and 2.15‐fold increase, respectively) (Figure 1B 1,F), whereas plasma miR‐134‐5p, miR‐223‐3p and miR‐765 were not obviously changed. Hence, miR‐29a‐3p, miR‐574‐3p and miR‐574‐5p were selected for further study.

### 3.3 Correlation analysis

**Figure 2:**
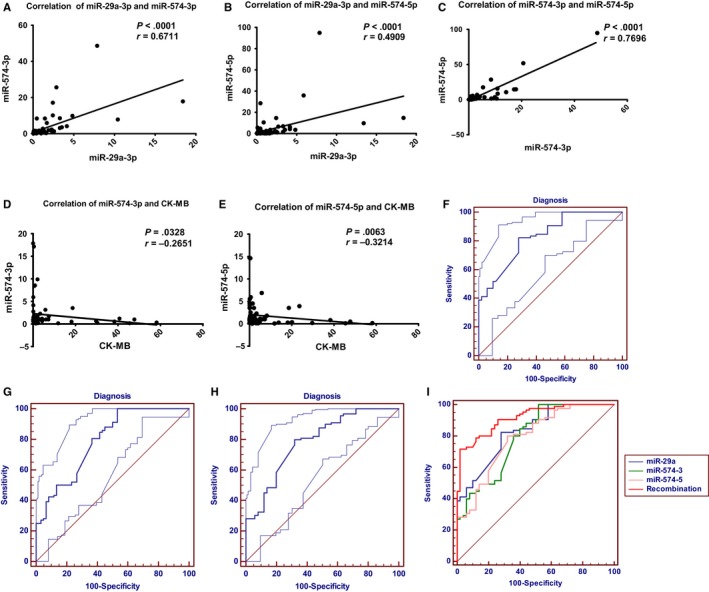
Correlation analysis and diagnostic value analysis. A, Correlation of miR‐29a‐3p and miR‐574‐3p in CAD patients. B, Correlation of miR‐29a‐3p and miR‐574‐5p in CAD patients. C, Correlation of miR‐574‐3p and miR‐574‐5p in CAD patients. D, Correlation of CK‐MB and miR‐574‐3p. E, Correlation of CK‐MB and miR‐574‐5p. F, ROC curve of miR‐29a‐3p, control patients vs. CAD patients. G, ROC curve of miR‐574‐3p, control patients vs. CAD patients. H, ROC curve of miR‐574‐5p, control patients vs. CAD patients. I, Diagnostic value analysis of circulating miRNAs by ROC curve. The figures depict calculated ROC curve of three circulating miRNAs and the combined ROC curve

The three selected plasma miRNAs showed different distribution among all patients. We identified the correlation of the three selected plasma miRNAs in both CAD patients and control patients. They were strongly associated with each other in CAD patients with *P* < .0001 (miR‐29a‐3p and miR‐574‐3p: *r* = .6711; miR‐29a‐3p and miR‐574‐5p: *r* = .4909; miR‐574‐3p and miR‐574‐5p: *r* = .7696) (Figure 2A‐C). In control patients, miR‐574‐3p was related to both miR‐29a‐3p and miR‐574‐5p (*P* < .0001, *r* = .6256; *P* = .0133, *r* = .4118) (Figure S1A,B), whereas miR‐29a‐3p and miR‐574‐5p exhibited no correlation (Figure S1C).

We investigated the relationship between plasma miRNAs and two biological indicators (CK‐MB and hsTnT). None of these miRNAs were correlated with hsTnT. miR‐29a‐3p had no correlation with CK‐MB. miR‐574‐3p and miR‐574‐5p were negatively associated with CK‐MB (*P* = .0328, *r *= −.2651; *P* = .0063, *r *= −.3214) (Figure 2D,E).

### 3.4 Diagnostic potential of plasma miRNAs by ROC analysis

The ROC curves of miR‐29a‐3p, miR‐574‐3p and miR‐574‐5p revealed the probability of them as valuable biomarkers with AUCs of 0.830, 0.792 and 0.789, respectively (Figure 2F‐H). The related data are briefly summarized in Table S2.

As shown in Figure 2, miR‐29a‐3p, miR‐574‐3p and miR‐574‐5p were significantly correlated with each other, indicating the value of joint diagnosis. The combined AUC value was 0.916 (Figure 2I; Table S2), much higher than each single miRNA, suggesting the highest discriminatory power.

## 4 DISCUSSION

CAD seriously endangers human life and brings heavy economic burden to the society. A rapid and accurate diagnosis enables more effective treatment to block the development of CAD and potentially reduce mortality. Circulating miRNAs have been demonstrated as novel diagnostic biomarkers for CAD.

miR‐29a remarkably improves the endothelial function in human T2DM arterioles and facilitates the restoration of endothelium‐dependent vasodilation in resistance arterioles.^6^ miR‐29a negatively regulates the expression of myeloid cell leukaemia 1 (MCL‐1), a prosurvival protein indispensable for the survival and function of cardiomyocytes and VSMCs.^7^, ^8^ miR‐574‐5p and miR‐574‐3p are generated from the 5’ and 3’ arms of its pre‐miRNA precursor. miRNA‐574‐5p and miR‐574‐3p have been found to be co‐expressed and regulate physiological processes, such as cancer progression 14, 15, 16 and cardiovascular diseases.^11^ Up till now, most research on miR‐574‐3p focused on tumorigenesis 15, 16 and its role on CAD has not been illustrated.

In this study, we explored the diagnostic potential of circulating miR‐29a‐3p, miR‐574‐3p and miR‐574‐5p. Our data firstly identified the elevated expression of plasma miR‐573‐3p in CAD patients compared with control patients. We found the correlation of these three circulating miRNAs, indicating their combinative value. Each of them showed considerable diagnostic power from the AUC values of ROC analysis. Furthermore, their recombination exhibited much higher AUC value than each single miRNA.

The present study has several limitations. The number of experimental samples was insufficient. Large‐scale studies will be performed in the near future. Further experimental studies are needed to explore the mechanisms of up‐regulation of miR‐29a‐3p, miR‐574‐3p and miR‐574‐5p. The qRT‐PCR method is expensive and time‐consuming. Therefore, cheaper and faster techniques are expected to be developed in the near future.

In summary, our study addressed the diagnostic value of miR‐29a‐3p, miR‐574‐3p and miR‐574‐5p and they could be combined into a probe system to provide more efficient, sensitive and non‐invasive diagnosis for CAD than individual miRNA.

## CONFLICT OF INTEREST

The authors declare that there is no conflict of interest.

## AUTHOR CONTRIBUTIONS

PFL and L.Z designed the study; L.Z, Y.Z, S.X and H.D performed the laboratory experiments, analysed the data and made the graphics; L.Z wrote the initial draft; Yu.W and HZQ contributed essential reagents or tools; Yin.W revised and edited the manuscript; and WJZ collected the clinical data. All authors read and approved the final manuscript.

## Data Availability

The data used to support this study are within the article and its Supporting Information files.
