# Supplementary figures and images for: Clinical significance of circulating microRNAs as diagnostic biomarkers for coronary artery disease
Source: J Cell Mol Med. 2019 Nov 11;24(1):1146–50. doi: 10.1111/jcmm.14802 (PMC6933363; doi:10.1111/jcmm.14802)

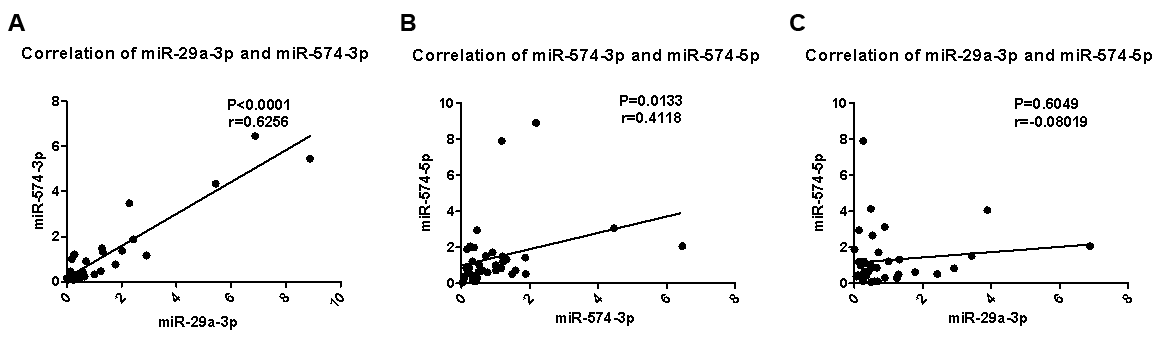

Supplement: Supplementary file 1 [file JCMM-24-1146-s001.tif]
