# Supplementary material for: Clinical significance of circulating microRNAs as diagnostic biomarkers for coronary artery disease
Source: J Cell Mol Med. 2019 Nov 11;24(1):1146–50. doi: 10.1111/jcmm.14802 (PMC6933363; doi:10.1111/jcmm.14802)

# Supplemental Tables

**Supplemental Table 1 The sequence information of miRNAs and primers**


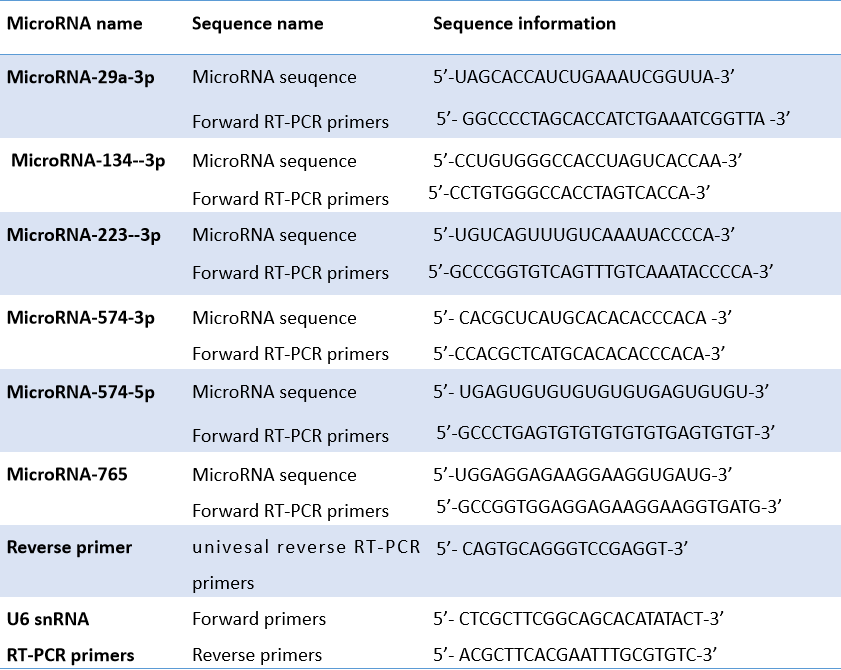


**Supplemental Table 2 Receiver operator characteristic curve (ROC) analysis of miRNA ratios**


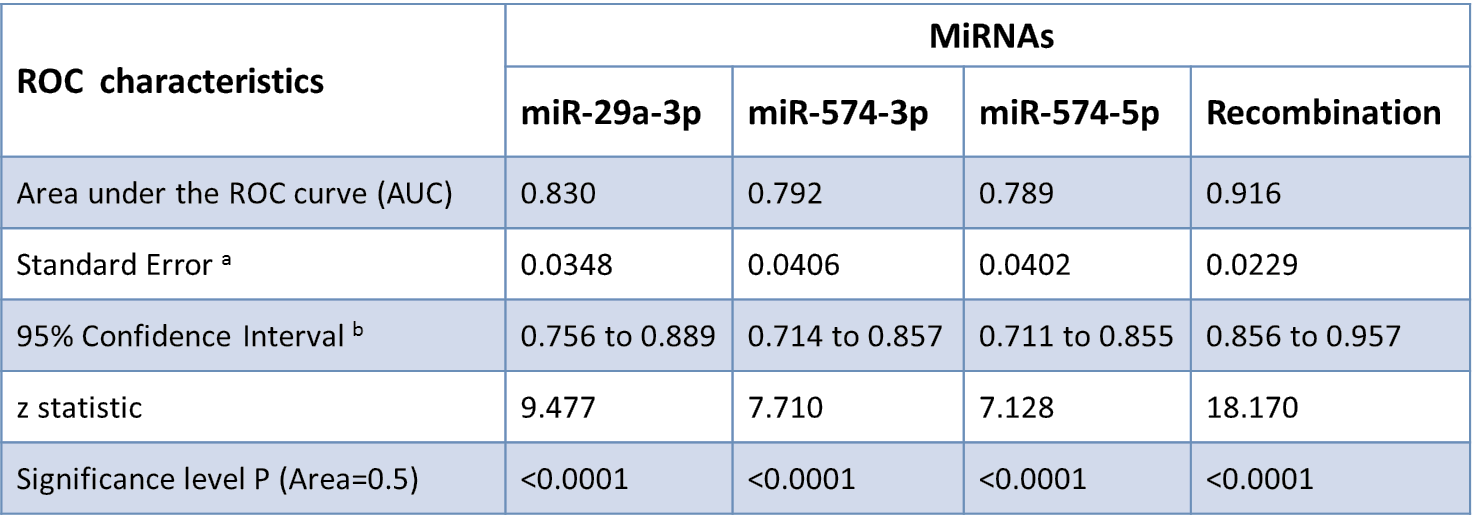

Supplement: Supplementary file 2 [file JCMM-24-1146-s002.docx]
